# Self-Reported Health and Well-Being Across Heterogeneous Groups of Caregivers

**DOI:** 10.1093/geroni/igaa057.2273

**Published:** 2020-12-16

**Authors:** Orla Sheehan, Jin Huang

**Affiliations:** Johns Hopkins University School of Medicine, Baltimore, Maryland, United States

## Abstract

Using the Caregiving Transitions Study (CTS) we compared the effects of caregiving on self-reported health and well-being in caregivers reporting providing dementia care, different levels of strain and amount of care provided. Caregivers (n-251) were 65% female, 36% African American and had a mean age of 71.8 years. A quarter of CGs reported being under a lot of strain and 47% provided care for persons with dementia. Dementia CGs (n=117) provided more hours of care per week (49.7 versus 37.7, p=0.001), more commonly reported high strain (36.8% versus 15.7%, p<0.03) and were more than twice as likely as non-dementia caregivers to report that caregiving interfered with taking care of their own health (33.9% versus 15.4%, p=0.003). Additional results will be reported on how these factors of dementia caregiving, level of strain, and hours of care affect well-being including perceived stress, treatment burden, depressive symptoms and health-related quality of life.

